# Identification of thiostrepton as a pharmacological approach to rescue misfolded alpha-sarcoglycan mutant proteins from degradation

**DOI:** 10.1038/s41598-019-43399-w

**Published:** 2019-05-06

**Authors:** Lucile Hoch, Sara F. Henriques, Celine Bruge, Justine Marsolier, Manon Benabides, Nathalie Bourg, Johana Tournois, Gurvan Mahé, Lise Morizur, Margot Jarrige, Anne Bigot, Isabelle Richard, Xavier Nissan

**Affiliations:** 1 CECS, I-Stem, 91100 Corbeil-Essonnes, France; 2 INSERM U861, I-Stem, 91100 Corbeil-Essonnes, France; 3 UEVE U861, I-Stem, 91100 Corbeil-Essonnes, France; 4 INTEGRARE, Genethon, Inserm, Univ Evry, Université Paris-Saclay, Evry, France; 5 Institut de Myologie, INSERM U974, Sorbonne Université, Paris, France

**Keywords:** High-throughput screening, Diseases

## Abstract

Limb-girdle muscular dystrophy type 2D (LGMD2D) is characterized by a progressive proximal muscle weakness. LGMD2D is caused by mutations in the gene encoding α-sarcoglycan (α-SG), a dystrophin-associated glycoprotein that plays a key role in the maintenance of sarcolemma integrity in striated muscles. We report here on the development of a new *in vitro* high-throughput screening assay that allows the monitoring of the proper localization of the most prevalent mutant form of α-SG (R77C substitution). Using this assay, we screened a library of 2560 FDA-approved drugs and bioactive compounds and identified thiostrepton, a cyclic antibiotic, as a potential drug to repurpose for LGMD2D treatment. Characterization of the thiostrepton effect revealed a positive impact on R77C-α-SG and other missense mutant protein localization (R34H, I124T, V247M) in fibroblasts overexpressing these proteins. Finally, further investigations of the molecular mechanisms of action of the compound revealed an inhibition of the chymotrypsin-like activity of the proteasome 24 h after thiostrepton treatment and a synergistic effect with bortezomib, an FDA-approved proteasome inhibitor. This study reports on the first *in vitro* model for LGMD2D that is compatible with high-throughput screening and proposes a new therapeutic option for LGMD2D caused by missense mutations of α-SG.

## Introduction

Limb Girdle Muscular Dystrophies (LGMD) are a heterogeneous group of muscular dystrophies sharing common clinical presentation of weakness affecting the shoulder and pelvic girdles^1^. Within this group, sarcoglycanopathies are a subgroup caused by mutations in genes encoding the transmembrane protein sarcoglycan (SG) complex, located in the sarcolemma of striated muscles^2^. Because the SG complex plays a key role in the maintenance of sarcolemma integrity during muscle contraction, mutations in these genes lead to muscular dysfunction^3^. Four SGs, namely, α−, β−, γ−, δ−SG, have been described to cause different subtypes of LGMD, LGMD2D^4^, LGMD2E^5,6^, LGMD2C^7^, LGMD2F^8^, respectively. While the clinical features of these LGMDs are well described, the molecular mechanisms associated with sarcoglycanopathies remain poorly understood^9,10^. Analyses of large cohorts of patients have revealed that hundreds of different missense or null mutations of these genes can lead to sarcoglycanopathies^11^, with the most frequent type being the arginine-to-cysteine substitution at the 77th amino acid (R77C) in α-SG^12^. This mutation leads to the production of a misfolded but still functional protein, recognized by the endoplasmic reticulum quality control (ERQC) system and degraded by the endoplasmic-reticulum-associated protein degradation (ERAD) pathway through the ubiquitin-proteasome system^13,14^.

Although no treatment is currently available for LGMD2D, recent studies suggest that inhibition of the different steps in the endoplasmic reticulum (ER) degradation system could be effective in restoring mutated α-SG expression in the plasma membrane^13–15^. Evidence in support of this strategy has been described by our group and others, as revealed by the positive impact of kifunensine when targeting mannosidase I activity^13,15^ or the use of the proteasome inhibitor MG132^14^. More recent studies on correctors of the misfolded ∆F508 mutant protein in the cystic fibrosis transmembrane conductance regulator (CFTR) have described their positive impact on α-SG missense mutant protein localization in the plasma membrane in primary muscle cells from patients^16^. Together, these studies provide strong evidence that targeting cellular quality control or degradation pathways are of interest to rescue missense mutations that cause LGMD2D and encourage further investigations in the search for new pharmacological molecules.

High-throughput screening (HTS) has been commonly used for decades to repurpose drugs with good safety profiles for new therapeutic applications^17^. Conversely, there are no reported cellular models for LGMD that are compatible with the robustness and reproducibility constraints imposed on this technique. In the current study, we generated and validated an R77C-α-SG mCherry fusion protein, leading to the development of a new *in vitro* cellular model for LGMD2D. Taking advantage of this model, we screened 2560 compounds, including 1280 off-patent small molecules, of which 95% are approved drugs, and 1280 annotated bio-active molecules, for their capacity to restore the expression of this α-SG mutant form in the plasma membrane. One of the compounds was validated on secondary tests on the R77C substitution as well as on additional SG mutations opening a new avenue toward treatment of LGMD2D patients.

## Results

### Validation of the α-SGmCh fusion construct and characterization of the R77C mutant cellular model

**Figure 1:**
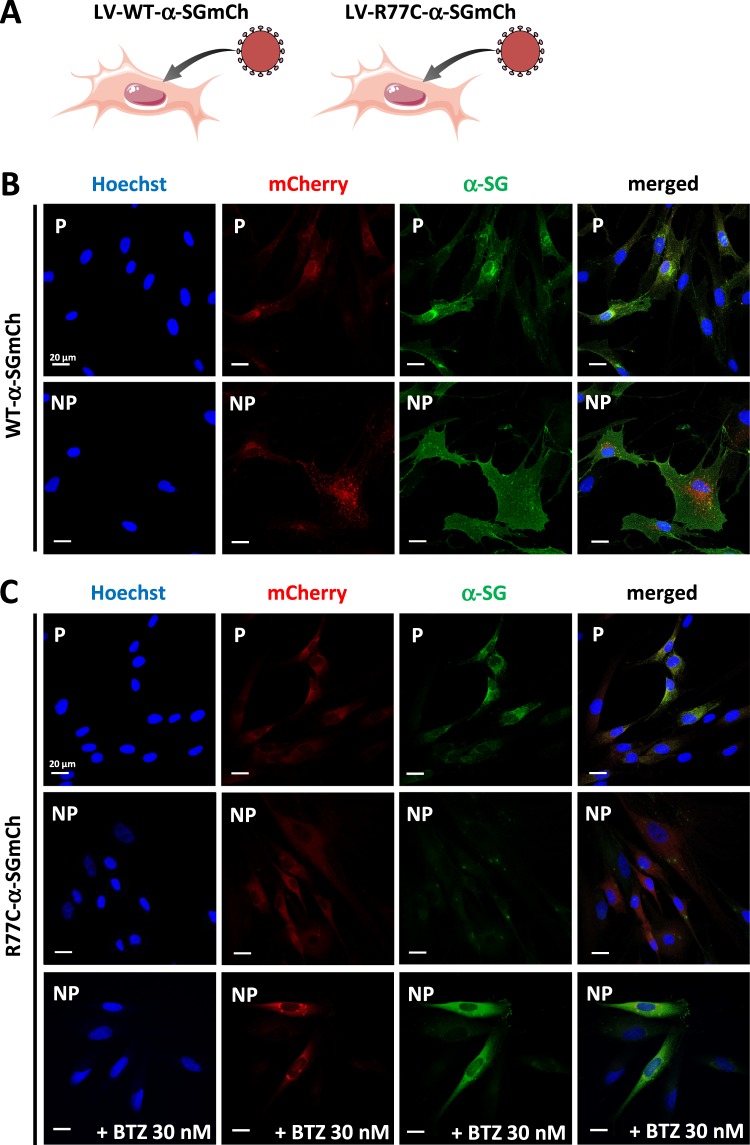
Characterization of α-sarcoglycan WT and R77C fusion constructs and effect of bortezomib treatment. (**A**) Schematic representation of the cellular models showing that immortalized fibroblasts from a LGMD2D patient carrying the R77C homozygous mutation were transduced with lentivirus expressing WT-α-SGmCh or R77C-α-SGmCh constructs. (**B**) Confocal images of mCherry signal (red) and α-SG (green) detected by immunofluorescence in fibroblasts transduced with the lentivirus expressing WT-α-SGmCh under permeabilized (P) and non-permeabilized condition (NP). (**C**) Confocal images of mCherry signal (red) and α-SG (green) detected by immunofluorescence in fibroblasts transduced with the lentivirus expressing R77C-α-SGmCh under permeabilized (P) and non-permeabilized condition (NP) and following bortezomib (BTZ) treatment at 30 nM. Nuclei are labelled by Hoechst staining (blue). Scale bar = 20 µm.

In order to easily evaluate the membrane localization of the wildtype (WT) and R77C mutant α-SG in the heterologous condition, a system was generated that allowed the simultaneous identification of the positive cells for the exogenous protein and the quantification of the protein localization in the cell membrane compartment. A fusion protein was created, consisting of the coding sequence for human WT or R77C-α-SG fused with a mCherry (mCh) fluorescent reporter at the C-terminus. A flexible linker was inserted between the two coding sequences to avoid protein interference in protein maturation and folding. The constructs, hereafter termed WT-α-SGmCh and R77C-α-SGmCh, were placed under the transcriptional control of the cytomegalovirus (CMV) promoter and inserted into a lentivirus backbone (Supplementary Fig. 1A). Immortalized fibroblasts from a LGMD2D patient homozygous for R77C were used as cellular model for screening because of their absence of endogenous α-SG. WT-α-SGmCh lentivirus was transduced with a multiplicity of infection (MOI) of 20 in these cells and monitored by evaluating the mCh fluorescence signal (Fig. 1A,B, top panels). The integrity of the fused protein was confirmed by showing co-localization of the mCh signal (red) and α-SG, as detected with an α-SG antibody (NCL-L-a-SARC) directed against the extracellular domain of α-SG (green) in a permeabilized condition (Fig. 1B, top panels). Immunofluorescence (IF) staining was then performed with the same α-SG antibody in a non-permeabilized condition, indicating that the fused protein was properly located at the cell membrane even in the absence of the other sarcoglycans as it was previously observed on other cell types^14,16^. A presence positive staining of α-SG was only detected in with mCh signal (Fig. 1B, bottom panels), confirming that the membrane protein revealed by IF was produced by the exogenous SGCA sequence.

The R77C-α-SGmCh fusion protein was characterized after validation of the WT-α-SGmCh construct. The choice of this particular mutation was based on the number of occurrences of patients affected with this mutation that were reported in the Leiden Muscular Dystrophy database (http://www.dmd.nl) and on previous studies demonstrating the rescue of the R77C protein using pharmacological treatments^13–16,18^. Confocal analysis of α-SG IF revealed intracellular staining in the permeabilized condition (Fig. 1C, top panels) and no detectable membrane localization of the R77C mutant protein in the non-permeabilized condition (Fig. 1C, middle panels), indicating retention of the protein. Further investigations of α-SG subcellular localization, with staining of the ER based on the recognition of the KDEL signal sequence, confirmed the ER retention of the R77C mutant protein (Supplementary Fig. 1B). Finally, the relevance of the screening model was validated by showing the rescue of R77C α-SG localization in the plasma membrane, following a pharmacological proteasome inhibition with bortezomib (BTZ) at 30 nM (Fig. 1C, bottom panels).

### High-content screening assay development and optimization

**Figure 2:**
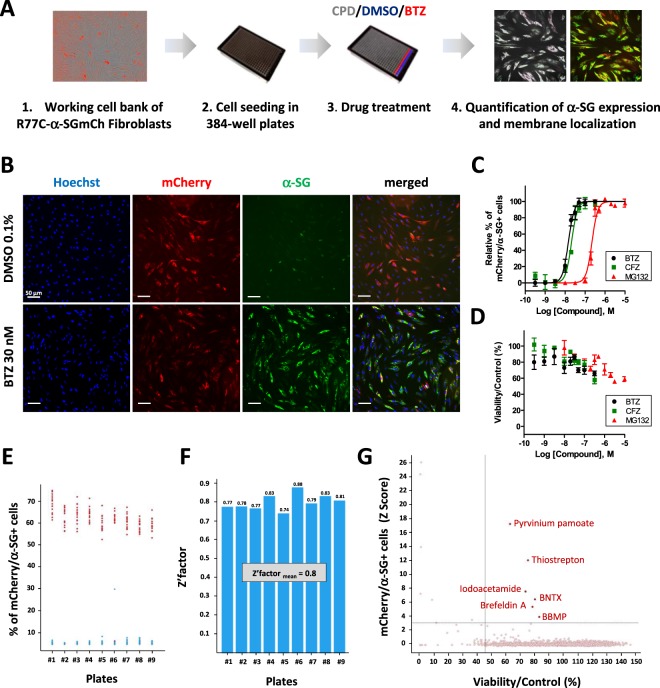
High-content screening for R77C-α-SGmCh membrane rescue. (**A**) Workflow for the high-content screening of R77C-α-SGmCh membrane rescue expression in 384 well plates. CPD = tested compounds. (**B**) mCherry fluorescent signal (red) and α-SG staining (green) in non permeabilized condition in fibroblasts overexpressing R77C-α-SGmCh treated with 0.1% DMSO and 30 nM bortezomib. Nuclei are labelled by Hoechst staining (blue). Scale bar = 50 µm. (**C**,**D**) Quantification of mCherry and membrane α-SG positive fibroblasts (**C**) and cell viability (**D**) following treatment with increasing concentrations of three proteasome inhibitors; bortezomib (black), carfilzomib (green) and MG132 (red). Values are expressed as percentage of the maximal response induced by bortezomib (**C**) or as percentage of the response induced by 0.1% DMSO (**D**) and each point represents the mean ± SD of four replicates. (**E**) High-throughput screening validation for the R77C-α-SGmCh membrane rescue in fibroblasts treated with the negative control, 0.1% DMSO, and the positive control, 30 nM bortezomib, in each of the 384-well plates of the screening. (**F**) Determination of the Z′ factor for each in each of the 384-well plates of the screening. (**G**) Primary screen cell-based assay for R77C-α-SGmCh membrane rescue. Dot plot representation of the effects of the 2560 drugs on R77C-α-SGmCh membrane expression (Z score $>$3) and cell viability (Viability $>$45%).; thiostrepton = THSP; bortezomib = BTZ, carfilzomib = CFZ.

In order to quantify the level of α-SG in the plasma membrane through high-content imaging, a working cell bank of LGMD2D patient fibroblasts that had been previously stably transduced with the R77C-α-SGmCh lentivirus was prepared and the process for HTS was set up as outlined in Fig. 2A. All experiments were carried out in 384-well plates. Immunostaining was conducted under the non-permeabilized condition using the α-SG antibody, 24 h after treatment with either 0.1% DMSO as the negative control or 30 nM bortezomib as the positive control. Images were acquired using the automated imaging CellInsight CX7 HCS Platform. Masks and algorithms were developed with the HCS Reader software to define the nuclear (Hoechst), membrane α-SG and mCherry fluorescent signals (Supplementary Fig. 2A). Quantification of cells positive for α-SG membrane staining and the mCherry signal was then carried out for the negative and positive controls. Robustness of the assay was validated through calculation of a Z’ factor of 0.78 in 5 independent plates, with 32 replicates of negative and positive controls in each plate (Supplementary Fig. 2B). Finally, dose response experiment using three known proteasome inhibitors, bortezomib, carfilzomib (CFZ) and MG132 was performed (Fig. 2B,C), confirming a dose dependent effect of these drugs on R77C α-SG rescue. Quantification of α-SG staining indicated that among these three drugs, the most efficient treatment was bortezomib, inducing a rescue of the membrane staining with an EC_50_ of 15 nM and reaching maximum efficacy at 30 nM. Carfilzomib and MG132 were also potent drugs for R77C-α-SG mutant rescue, with an EC_50_ of 21 nM and 222 nM, respectively (Fig. 2C). The effect of these drugs on cell viability was then measured by counting the number of cells 24 h after treatment, showing a decrease of 30% to 40% of cells as detected by Hoechst at doses corresponding to the EC_50_ for the three inhibitors (Fig. 2D).

### Identification of a new inhibitor of misfolded R77C-α-SG protein degradation using high-throughput screening

**Figure 3:**
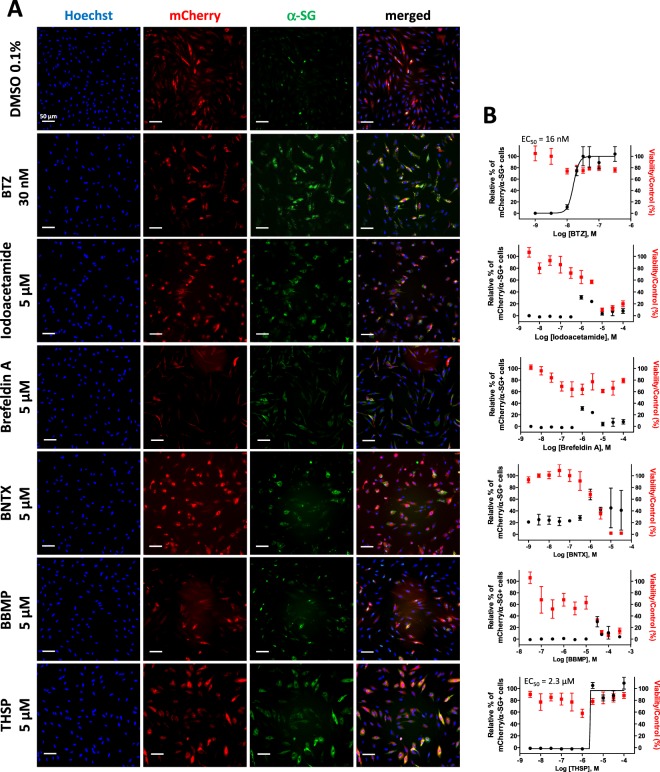
Effect of the five identified compounds on membrane localization of R77C α-SG protein. (**A**) mCherry fluorescent signal (in red) and α-SG staining (in green) in non permeabilized condition in fibroblasts overexpressing R77C-α-SGmCh treated with 0.1% DMSO, bortezomib 30 nM and the five drugs identified at the dose of 5 µM (iodoacetamide, brefeldin A, BNTX, BBMP and thiostrepton). Nuclei are labelled by Hoechst staining (blue). Scale bar = 50 µm. (**B**) Quantification of mCherry and membrane α-SG positive cells and cell viability following treatment with increasing concentrations of bortezomib, iodoacetamide, brefeldin A, BNTX, BBMP and thiostrepton. Values are expressed as the percentage of the maximal response induced by bortezomib and each point represents the mean ± SD of four replicates; thiostrepton = THSP; bortezomib = BTZ.

HTS was then performed with the aim of finding new compounds capable of the membrane localization of R77C-α-SGmCh, with potentially lower toxicity than the 3 proteasome inhibitors that had been tested. A total of 2560 compounds, comprising 1280 bio-active compounds from the Library of Pharmacologically Active Compounds (LOPAC) and 1280 approved drugs from Prestwick Chemical library, were tested at 5 µM. Screening quality was ensured by a statistically relevant difference between the negative and positive controls, giving a mean Z’ factor of 0.8 (Fig. 2E,F). Compounds were considered as potential candidates when their effect was superior to three standard deviations from the mean for all the tested compounds, without affecting cell viability by more than 55%. This led to an initial list of six hits, thiostrepton (THSP), idoacetamide, 7-benzylidenenaltrexone (BNTX), brefeldin A, 5-benzylsulfonyl-4-bromo-2-methylpyridazin-3-one (BBMP) (Fig. 2G and Supplementary Table 1) and pyrvinium pamoate, the latter being discarded because of artefactual fluorescence (Supplementary Fig. 3). Retest experiments were conducted with increasing concentrations from the nanomolar to micromolar range for the five primary hits (Fig. 3). Of these, only THSP, a natural cyclic oligopeptide antibiotic approved in veterinary medicine for the topical treatment of dermatologic infections, was validated as an hit of interest, with an EC_50_ of 2.3 µM and a toxicity of only 20%. Of note, THSP induced a steep dose-response curve, with a threshold dose of 1 µM and maximum effect at 3 µM.

### Characterization of thiostrepton’s mode of action

**Figure 4:**
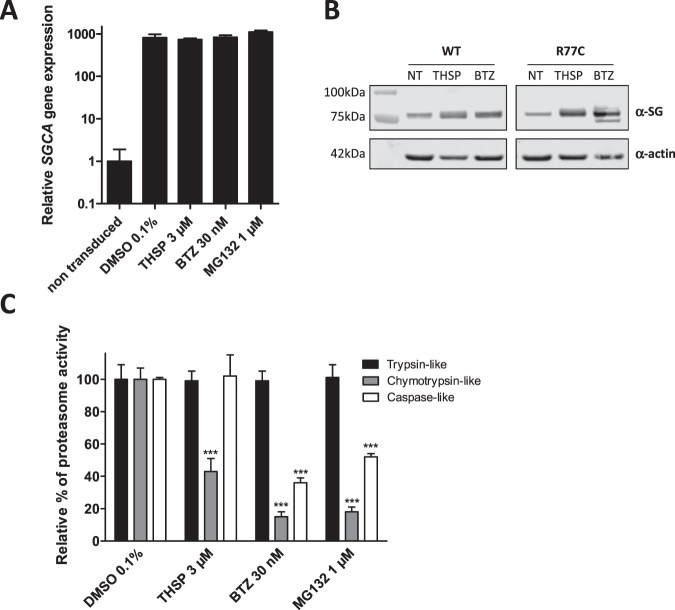
Mechanism of action of Thiostrepton. (**A**) Measure of *SGCA* gene expression by qPCR in fibroblasts overexpressing R77C-α-SGmCh and treated with 0.1% DMSO, 3 µM thiostrepton, 30 nM bortezomib or 1 µM MG132 for 24 hours. Data are normalized to non-transduced fibroblasts. (**B**) Grouping of western blots analysis of α-SG and α-actin expression in non-treated (NT) fibroblasts overexpressing WT- and R77C-α-SGmCh or treated with thiostrepton (THSP, 3 µM) or bortezomib (BTZ, 30 nM) for 24 hours. Full-length blots are presented in Supplementary Fig. 4. (**C**) Quantification of trypsin-like, chymotrypsin-like and caspase-like activities of the proteasome in fibroblasts overexpressing R77C-α-SGmCh treated for 24 h with 0.1% DMSO, 3 µM thiostrepton, 30 nM bortezomib or 1 µM MG132.Values are expressed as the percentage of the response relative to 0.1% DMSO and each point represents the mean ± SD of four replicates.

In order to determine whether the effect of THSP on R77C-α-SG mutant protein is transcriptional or post-transcriptional in origin, the *SGCA* gene expression was evaluated after THSP (3 µM) treatment, using bortezomib (30 nM) and MG132 (1 µM) (Fig. 4A). No significant difference was observed following these treatments in comparison to 0.1% DMSO, indicating no transcriptional change in the *SGCA* gene after treatment. α-SG protein levels were then investigated after THSP (3 µM) or bortezomib (30 nM) treatment by immunoblot analysis (Fig. 4B and Supplementary Fig. 4). Increased α-SG protein levels were detected in fibroblasts overexpressing WT- and R77C-α-SGmCh after THSP and bortezomib treatment. This analysis showed also the presence of an additional and lower band in cells treated with BTZ. An enzymatic treatment removing N-linked oligosaccharides indicated that this additional band corresponds to a non-glycosylated form of α-SG, indicating that the BTZ treatment leads to a certain level of immature α-SG, an event which is not seen with THSP (Supplementary Fig. 5). Interestingly, THSP has been previously identified as an inhibitor of FOXM1 protein expression through a proteasome-dependent mechanism^19^ and has been reported to interact covalently with the regulatory particle of triple-ATPase (Rpt) subunits in the 19 S proteasome^20^. We therefore evaluated the different proteasomal activities after treatment with THSP. At the 3 µM concentration, THSP partially inhibited the chymotrypsin-like activity of the proteasome, while bortezomib (30 nM) and MG132 (1 µM) blocked both the chymotrypsin-like and caspase-like activities, suggesting a different mode of action for these compounds on the proteasome (Fig. 4C).

**Figure 5:**
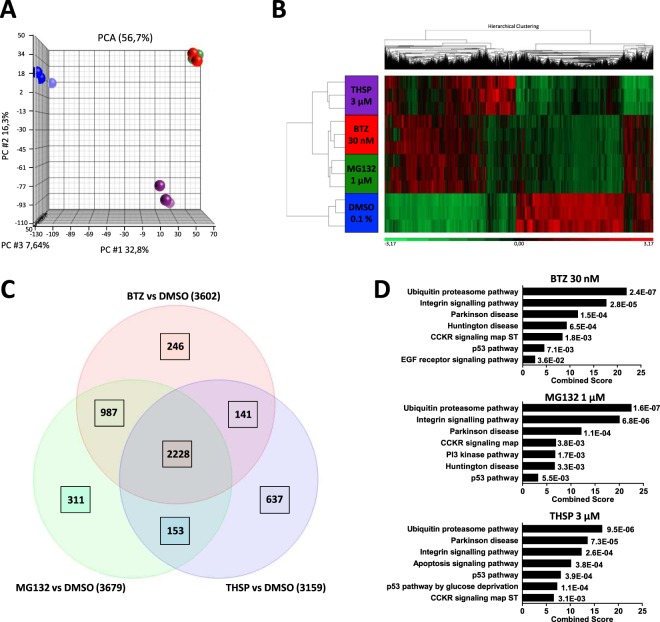
Transcriptomic analysis. (**A**) The PCA graph of global gene expression data computed using Partek Genomic Suite and Partek Flow. 0.1% DMSO samples are shown as blue spheres; 3 µM thiostrepton samples in purple; 30 nM bortezomib samples in red and 1 µM MG132 samples in green. (**B**) Hierarchical clustering and heat map of significantly expressed genes in thiostrepton 3 µM vs 0.1% DMSO using RNA sequencing in fibroblasts overexpressing R77C-α-SGmCh. Color coding from green to red depicts gene expression differences between treatments from low to high expression, respectively. (**C**) Venn diagram illustrating the number of shared genes modulated in fibroblasts overexpressing R77C-α-SGmCh after 24 h of treatment with 0.1% DMSO, 3 µM thiostrepton, 30 nM bortezomib or 1 µM MG132. (**D**) Functional enrichment analysis of up-regulated genes in fibroblasts overexpressing R77C-α-SGmCh after 24 h of treatment with 30 nM bortezomib, 3 µM thiostrepton or 1 µM MG132 vs 0.1% DMSO. Analysis has been performed with the Panther pathway database. Values are expressed as combined scores and p-values are indicated; thiostrepton = THSP; bortezomib = BTZ.

We then conducted an RNA-seq analysis using the Ion AmpliSeq Transcriptome Human Gene Expression core panel to characterize the transcriptomic changes that occur after treatment with 3 µM THSP, 30 nM bortezomib and 1 µM MG132, when compared with 0.1% DMSO. We generated an average of 11 million pairs of reads per sample. RNA-seq-based gene expression levels were calculated from reads per million (RPM) values. The principal component analysis (PCA) revealed a clear separation between untreated and treated samples. In addition, treatments with 30 nM bortezomib and 1 µM MG132 were grouped in the same cluster, suggesting a common transcriptome signature for these two drugs (Fig. 5A). An unsupervised hierarchical clustering was performed to assess similarities and changes in global gene expression profiles between the different treatments, again indicating the similarity between 30 nM bortezomib and 1 µM MG132 (Fig. 5B) and showing that THSP is closer to these treatments than to the DMSO condition. Statistical differential expression analysis comparing 0.1% DMSO and 3 µM THSP (false discovery rate (FDR) < 0.01) revealed that 3159 genes were significantly differentially expressed, with 1577 genes being up‐regulated and 1582 genes down‐regulated. Among these 3159 genes, 2228 were modulated by all the treatments, suggesting common consequences of these drugs (Fig. 5C). Moreover, Panther pathway analysis using the Enrichr web interface suggested that these three different treatments were commonly enriched for major signaling pathways, the main one being the ubiquitin-proteasome pathway (Fig. 5D) as expected. Nevertheless, this analysis revealed that 637 genes were specifically modulated by 3 µM THSP for which Panther analysis revealed an enrichment of angiogenesis and p53 pathways in accordance with previous reports^21,22^ (Fig. 5C,D).

### Combinatorial effect of thiostrepton and bortezomib on R77C-α-SGmCh membrane rescue

**Figure 6:**
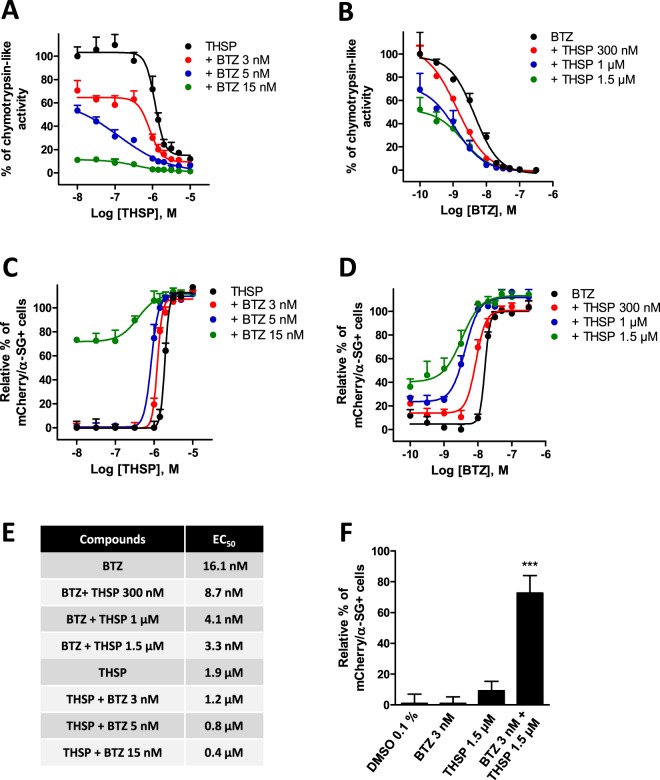
Synergistic effects of thiostrepton and bortezomib. Quantification of chymotrypsin-like activity of the proteasome (**A**,**B**) or mCherry and membrane α-SG expression (**C**,**D**) in fibroblasts overexpressing R77C-α-SGmCh. Cells were treated for 24 h with increasing concentration of thiostrepton in the presence of 3 nM, 5 nM or 15 nM bortezomib (**A**,**C**) or with increasing concentrations of bortezomib in the presence of 300 nM, 1 µM or 1.5 µM thiostrepton (**B**,**D**).Values are expressed as percentage of the response induced by 0.1% DMSO (**A**,**B**) or as percentage of the maximal response induced by bortezomib (**C**,**D**) and each point represents the mean ± SD of four replicates. (**E**) EC_50_ of thiostrepton, bortezomib and combination treatments on membrane α-SGmCh membrane rescue. (**F**) Quantification of mCherry and membrane α-SG positive cells after 0.1% DMSO, 1.5 µM thiostrepton, 3 nM bortezomib and combination treatments. ***p ≤ 0.001; thiostrepton = THSP; bortezomib = BTZ.

Since THSP and bortezomib had different effects on both proteasome activities and cell transcriptome, there was a potential interest in combining them for potentiation. We measured the chymotrypsin-like activity of the proteasome with increasing concentrations of THSP in the presence of 3 nM, 5 nM or 15 nM bortezomib in one hand and with increasing concentrations of bortezomib in the presence of 300 nM, 1 µM or 1.5 µM THSP in the other hand. Analysis of the dose-response curves revealed a higher inhibition of the chymotrypsin-like activity induced by THSP when combined with bortezomib than with the two drugs used alone (Fig. 6A,B). Quantification of the number of cells with membrane localization of this protein was carried out in the presence of increasing doses of these two drugs, either alone or in combination, to determine whether or not THSP and bortezomib might act synergistically on R77C-α-SG mutant rescue. Analysis of the dose-response curves revealed a shift to the left when the drugs are combined, in comparison to the individual drugs alone (Fig. 6C,D). Analysis of this set of experiments revealed a decrease in the EC_50_ obtained following drug combination treatments (3.3 nM and 0.4 µM, respectively), as compared to the individual drugs alone, i.e., 16.1 nM bortezomib and 1.9 µM THSP (Fig. 6E). Finally, this synergistic effect on R77C-α-SGmCh mutant protein rescue was confirmed by combining low doses of bortezomib (3 nM) and THSP (1.5 µM), showing a significantly higher effect of this combination than with the two drugs alone (Fig. 6F). Overall, these results provide evidence of a synergistic effect of THSP and bortezomib on R77C-α-SGmCh mutant protein rescue from proteasomal degradation.

### Rescue of the R77C-α-SG mutant in iPSC-derived myoblasts following thiostrepton treatment

**Figure 7:**
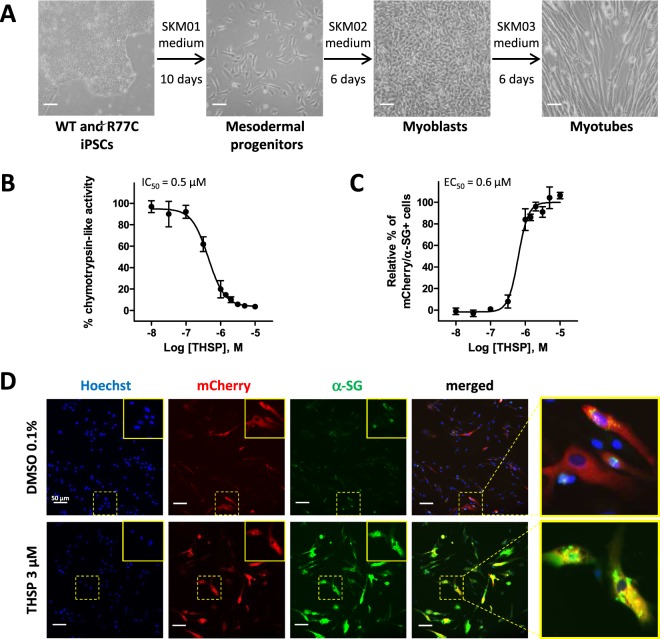
Thiostrepton effect on the rescue of R77C-α-SGmCh protein in iPSC-derived myoblasts. (**A**) Differentiation of iPSCs into myoblasts following a 3-step protocol of differentiation. iPSC-derived myoblasts were transduced with the lentivirus overexpressing R77C-α-SGmCh and treated for 24 h with increasing concentrations of thiostrepton or negative and positive controls (0.1% DMSO and 30 nM bortezomib, respectively). (**B**) Quantification of the chymotrypsin-like activity of the proteasome following thiostrepton 3 µM treatment. Values are expressed as the percentage of the response induced by DMSO (0.1%) (**C**) Quantification of mCherry and membrane α-SG positive cells after thiostrepton 3 µM treatment. Values are expressed as percentage of the maximal response induced by bortezomib. (**D**) Images of mCherry fluorescent signal (red), membrane α-SG (green) and Hoechst staining (blue) in iPSC-derived myoblasts treated with 0.1% DMSO or thiostrepton 3 µM in non-permeabilized condition. Outsets represent higher magnification images. Scale bar = 50 µm; thiostrepton = THSP; bortezomib = BTZ.

The efficacy of THSP in rescuing the R77C-α-SGmCh mutant protein was assessed on muscular cells to confirm the data collected on the fibroblast cellular model. To do so, WT and LGMD2D patient fibroblasts were reprogrammed into induced pluripotent stem cells (iPSCs) as no primary myoblasts carrying the homozygous R77C mutation were available. Following a full quality control of their pluripotency capacities, as revealed by the expression of OCT4, NANOG, SSEA4, TRA160 by IF or SSEA3, TRA181, based on flow cytometry and a high activity of alkaline phosphatase (Supplementary Fig. 6A–C), iPSCs were then differentiated into myoblasts and myotubes using a 3-step protocol of differentiation (Fig. 7A). Myoblasts and myotubes obtained using this protocol were characterized by monitoring the expression of the two myogenic markers, MyoD and MF20 (Supplementary Fig. 6D,E). While qPCR revealed high expression of *SGCA* mRNA (Supplementary Fig. 6F), the endogenous protein was not detected by IF, preventing its use for investigating the effect of THSP (Supplementary Fig. 6D,E). Myoblasts derived from LGMD2D patient iPSCs were therefore transduced with the lentivirus overexpressing R77C-α-SGmCh to evaluate the effect of THSP on muscular cells. Quantification of chymotrypsin-like activity in these cells revealed a dose-dependent effect of THSP, with an IC_50_ of 0.5 µM (Fig. 7B). Similarly, analysis of R77C-α-SGmCh protein localization revealed a rescue of this protein in the plasma membrane, with an EC_50_ of 0.6 µM (Fig. 7C,D).

### Rescue of different α-SG mutants through thiostrepton treatment

**Figure 8:**
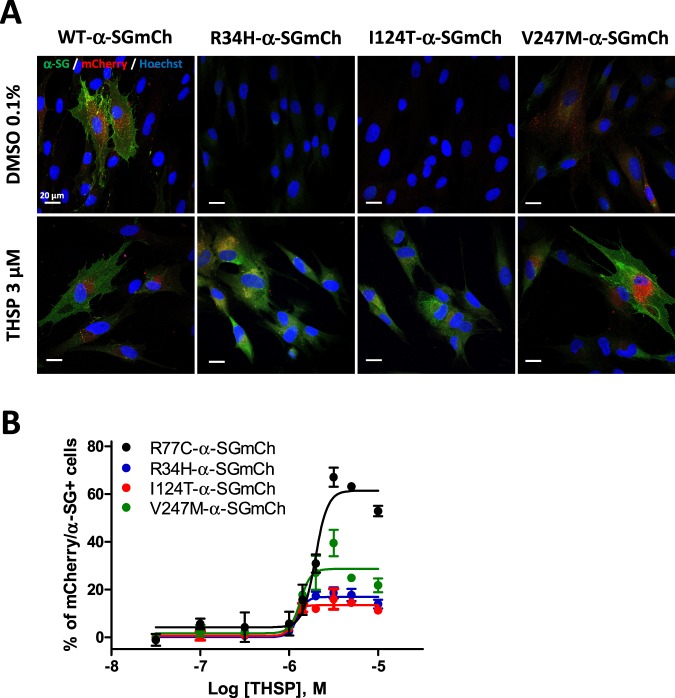
Evaluation of thiostrepton treatment on other missense α-SG mutants. Immortalized fibroblasts were transduced with lentivirus expressing WT-, R34H-, I124T- or V247M-α-SGmCh constructs and treated for 24 h with 0.1% DMSO or increasing concentrations of thiostrepton. The membrane α-SG expression was monitored by IF under non-permeabilized condition. (**A**) Confocal images of mCherry fluorescent signal (red), membrane α-SG (green) and Hoechst staining (blue) after 0.1% DMSO or THSP 3 µM treatments. Scale bar is 20 µm. (**B**) Quantification of mCherry and membrane α-SG positive cells following treatment with increasing concentrations of Thiostrepton. Values are expressed as percentage of mCherry and α-SG positive cells; thiostrepton = THSP; bortezomib = BTZ.

We then evaluated the effect of THSP on the rescue of other missense α-SG mutants. We selected three mutations with severe phenotypes (R34H, I124T and V247M) that are among the most frequent mutations in *SGCA* encoding for misfolded proteins that are not rescuable by kifunensine^15^. Lentiviruses expressing the R34H, I124T and V247M α-SGmCh mutants were generated following the same experimental strategy employed for the R77C mutant and were used to transduce the immortalized R77C patient fibroblasts. Confocal immunofluorescence analysis in non-permeabilized condition revealed the absence of detectable α-SG staining in the membrane compartment in all the mutant transduced cells (Fig. 8A, top panels). Analysis of R34H, I124T and V247M-α-SGmCh protein localization was then evaluated following treatment with 3 µM THSP and showed the rescued localization of the three mutant proteins at the membrane level (Fig. 8A, bottom panels). Finally, analysis of the dose-response curves revealed a similar effect of THSP, with an EC_50_ of 1.4 µM, on R77C, I124T, R34H and V247M α-SGmCh mutant proteins, but different maximum efficacies ranging between 15% and 60% of rescued cells (Fig. 8B).

## Discussion

In the current study, we established a HTS method that can be used to identify candidate molecules able to rescue α-SG mutant proteins from ER-retention and early degradation. Thanks to this platform, we identified THSP as a candidate compound to be explored as a new therapeutic molecule for the treatment of LGMD2D patients affected by missense mutations. Beyond the effect on proteasomal degradation of this specific compound, our results shed a new light on an experimental paradigm suitable for the treatment of other genetic conditions associated to missense mutations.

The rescue of ER-retained and prematurely degraded sarcoglycan mutant proteins has been previously demonstrated using pharmacological compounds^13–16,18^. Most of the compounds used on these studies were specifically chosen for their ability to target key players of the ERQC and ERAD pathways, the mechanisms involved in the active degradation of mutant proteins, or to rescue other misfolded proteins such as in the case of CFTR correctors^16^. Even though specific mechanisms can be involved in the recognition of the misfolding structure of each disease-causing protein, the above mentioned studies demonstrates that some common features make possible to repurpose drugs from one to the other disease^23^. Here, we performed a drug screening on the most frequent LGMD2D causing mutation (p.R77C) as a platform to identify drugs that would also benefit other misfolding diseases. As designed, our screening procedure allowed simultaneous testing for hundreds of possible repurposable drugs and bioactive compounds able to rescue the R77C α-SG mutant protein to the cell membrane compartment. α-SG is expressed in mature skeletal muscle fibers. To overcome the current inability to attain suitable muscle cellular models has hindered the use of endogenous mutant expression for drug identification, we have introduced a heterologous stable overexpression system in skin cells. Using this cellular model, as a proof of concept, we identified a promising new lead compound, THSP, for the rescue of ER-retained α-SG R77C mutant protein that was later confirmed to rescue other patient-derived mutant proteins, reinforcing the rationale to use the R77C mutation as point of entry.

THSP is a natural antibiotic produced by microorganisms in the genus Streptomyces discovered in 1955^24–26^. It is mainly used as an anti-parasitic veterinary product. Over the past decade, several studies have highlighted a new potential use in humans, based on its apoptotic effect on cancer cells^27,28^, alone or in combination with bortezomib^29^. Interestingly, normal cells were shown to be resistant to THSP-induced apoptosis through expression of WT p53 protein^21,27^, suggesting that its application in healthy tissues might be a safe strategy. Furthermore, THSP was recently proposed as a less toxic alternative to bortezomib for topical treatment for Psoriasis-like inflammation caused by TLR9 through, in particular, the attenuation of proteasomal degradation^30^. More recently, cell-based HTS has identified THSP as a regulator of Drosophilia inhibitor of apoptosis protein 1 (DIAP1) degradation through direct and irreversible inhibition of the 19S proteasome subunit^20^. Interestingly, our study is in line with these results as we demonstrated a robust effect of THSP on α-SG mutant degradation and proteasome activity, with an EC_50_ of 2.3 µM and 1.9 µM, respectively, both in the same range as in the DIAP1 study (EC_50_ of 4.9 µM). Our results also highlight the value of pharmacological action on proteasome activity to rescue misfolded α-SG proteins from degradation, as originally described for MG132^13,14^. Within the class of proteasome inhibitors, bortezomib has been described as one the most potent treatment, leading to its approval by the US FDA for the treatment of patients with relapsed refractory multiple myeloma^31^. Even though our study demonstrates that bortezomib treatment can efficiently rescue R77C-α-SGmCh protein degradation, overall, it reports that the combination of bortezomib and THSP significantly increases the effect of the treatment on the proteasome activity and the α-SG rescue.

While our overall results describe a potential new class of drug for the treatment of LGMD2D, further preclinical and clinical investigations are required before evaluating its efficacy in patients. Firstly, this is because THSP is a veterinarian drug with no human toxicology reports, requiring several complementary investigations in animal models in order to safeguard human administration. Secondly, THSP is mainly used through topical administration, requiring optimization of its administration route by, for example, using nanoparticles to target muscles and limit potential off-target side effects^32^. Even though clinical treatment may be some way off, our results describe a method for the identification of new treatments for hundreds of diseases involving misfolded proteins and hope for LGMD2D patients who remain without curative treatment.

## Materials and Methods

### Plasmid cloning and mutagenesis

The sarcoglycan fusion protein was designed based on the human *SGCA* consensus coding sequence (CCDS) found in the NCBI portal (Gene ID: 6442, CCDS number 45729.1) and the mCherry sequence: The linker, in the amino-acid form of -GGGGS-, was chosen as a flexible type linker that also increased stability/folding of the fusion proteins^33^. The fusion protein nucleotide sequence was synthesized by Genecust and cloned in a vector plasmid prepared for lentivirus production. The final construct, termed α-SGmCh, was driven by the cytomegalovirus (CMV) promoter. The R34H, R77C, I124T and V247M mutations were generated based on the 100GT > AC, 229C > T, 371T > C and 739G > A nucleotide changes of *SGCA* gene, respectively. Mutagenesis was performed using the QuikChange XL Site-Directed Mutagenesis kit (200516, Agilent) according to manufacturer’s instructions. The primers used to introduce the R34H, R77C, I124T and V247M mutations are listed in Supplementary Table 2.

### Cell Culture, transduction and pharmacological treatments

The R77C fibroblasts used in this study were isolated from a patient biopsy obtained by the Genethon’s Cell Bank. Informed consents were obtained from the parents of the patient included in this study, complying with the ethical guidelines of the institutions involved and with the legislation requirements of the country of origin. Fibroblasts were transduced with a pBABE-puro-based retroviral vector containing sequence encoding the catalytic subunit of human telomerase reverse transcriptase (hTERT) and then selected in the presence of puromycin (1 mg/ml) for 10 days, as previously described^34^. Experimental protocols were approved by the french minister of research: AC-2013-1868. Fibroblasts were cultured in Dulbecco’s modified Eagle’s medium + GlutaMAX (Invitrogen) supplemented with 10% fetal bovine serum (research grade, Sigma-Aldrich) and 1% Penicillin-Streptomycin (Invitrogen). For overexpression of the WT or mutated α-SG, immortalized fibroblasts were transduced with a lentiviral vector expressing human WT-, R77C-, R34H-, I124T- or V247M-α-SGmCh with a multiplicity of infection (MOI) of 20 in the presence of 4 µg/ml of polybrene (Sigma-Aldrich). Cells were seeded on plates coated with 50 µg/ml of collagen I and maintained in a humidified atmosphere of 5% CO_2_ at 37 °C. Twenty-four hours after seeding, cells were treated with 30 nM of bortezomib (Selleckchem) or the carrier 0.1% dimethyl sulfoxide (DMSO, VWR). Cells were analyzed after 24 hours of treatment.

### Pluripotent stem cell culture and differentiation

The R77C fibroblasts were reprogrammed to iPSCs using Yamanaka’s original method with OCT4, KLF4, SOX2, c-Myc, transferred using retroviral vectors^35^. WT and R77C iPSCs were grown in colonies on matrigel (Corning), seeded at 5000 cells/cm^2^ and cultured in iPS-Brew XF medium (Macs, Milteny Biotech). iPSCs were differentiated into myoblasts following a protocol developed by Geneabiocell® (Caron *et al*., 2016). Briefly, iPSCs were seeded at 3,500 cell/cm² in 6-well plates coated with collagen I and maintained in SKM01 medium (AMSBIO) for 10 days with a passage at day 7. Medium was then switched to SKM02 medium (AMSBIO) until freezing of the myoblasts at day 16. For overexpression of the WT or mutated α-SG, iPSC-derived myoblasts were transduced with a lentiviral vector expressing human WT or R77C-α-SGmCh with a multiplicity of infection (MOI) of 43 in the presence of 4 µg/ml of polybrene (Sigma-Aldrich).

### High throughput screening

The high-throughput screening was conducted on Biocell 1800 (Agilent). For this, 1600 R77C-α-SGmCh fibroblasts were seeded in 38 μl of culture medium into black 384-well clear bottom plates. Twenty-four hours after seeding, 2 μl of compounds from the chemical libraries were transferred in monoplicate into cell assay plates. In each plate, negative control (0.1% DMSO) and positive control (30 nM bortezomib) were added in columns 1–23 and 2–24, respectively. Plates were then incubated for 24 hours and processed for α-SG detection assay. Each of the 9 plates of the screening were fixed and stained with the specific anti-α-SG antibody using the optimized protocol under non-permeabilized condition. To prevent the discovery of toxic molecules, the number of cells was monitored in parallel by counting Hoechst stained cells per field and candidates showing mortality superior to 55% were excluded.

### α-SG localization cell-based assay

After 24 hours of drug treatment, cells were fixed in 4% paraformaldehyde (10 min, room temperature). Immunocytochemistry was performed in a phosphate-buffered saline (PBS) solution supplemented with 1% bovine serum albumin (BSA; Sigma) for blocking (1 hour, room temperature) and with a mouse anti-α-SG (NCL-L-a-SARC, Novocastra, Leica) for primary hybridization step (overnight, 4 °C). Cells were stained with a fluorophore-conjugated secondary anti-mouse antibody (Invitrogen; 1 hour, room temperature) and nuclei were visualized with Hoechst 33342 (Invitrogen). α-SG localization was analyzed with a CellInsight CX7 HCS Platform (Cellomics Inc). The first channel was used for nuclei identification, the second one for membrane α-SG staining identification and the third one for mCherry tag identification. Pictures were acquired with a 10x objective in high-resolution camera mode and were analyzed using the colocalization bioapplication quantifying positive cells for membrane α-SG staining and mCherry tag expression.

### Chemical library

The chemical libraries were obtained from Prestwick Chemical and Sigma-Aldrich for the LOPAC and distributed in 384 well plate format. The Prestwick Chemical library contains 1280 FDA approved drugs and the LOPAC library contains a collection of 1280 pharmacologically active compounds. Both libraries were tested at 5 μM.

### Data analysis

Data analysis of the screening was performed using a customized Hiscreen application (Discngine) connected to Spotfire software (Tibco Software Inc.). The robustness of the assay was evaluated by calculating for each plate the Zʹ factor on the percentage of mCherry/α-SG positive cells parameter as follows Zʹ = 1 − [3(SDP + SDN)/(MP − MN)] where MP and MN correspond to the means of the positive (30 nM bortezomib) and negative (0.1% DMSO) controls, respectively, and SDP and SDN correspond to their S.D. Raw data related to the percentage of mCherry/α-SG positive cells were normalized to the mean of positive and negative controls, which are defined as 100% and 0%, respectively. Raw data related to cell number per field were normalized to the mean of negative controls. Hit selection was performed using in parallel the number of standard deviations from the mean for each readout value (Z-score) calculated per plate individually or per run where all plate data were pooled. Only hits whose Z-score plate and/or Z-score run was $\geqslant 3$ and that did not decrease cell number by more than 55% compared to 0.1% DMSO condition were selected for subsequent validation step. Hits were then tested at gradual concentrations (1 nM–100 µM) for parallel exploration of their efficacy and toxicity.

### Quantitative PCR

Total RNAs were isolated using the RNeasy Mini extraction kit (Qiagen) according to the manufacturer’s protocol. A DNase I digestion was performed to avoid genomic DNA amplification. RNA levels and quality were checked using the NanoDrop technology. A total of 500 ng of RNA was used for reverse transcription using the SuperScript III reverse transcription kit (Invitrogen). Quantitative polymerase chain reaction (qPCR) analysis was performed using a QuantStudio 12 K Flex real-time PCR system (Applied biosystem) and Luminaris Probe qPCR Master Mix (Thermo Scientific), following the manufacturers’ instructions. α-SG expression analysis were performed using the TaqMan gene expression Master Mix (Roche), following the manufacturer’s protocol. Quantification of gene expression was based on the DeltaCt method and normalized on 18S expression (Assay HS_099999). Primer sequences for *SGCA* were: 5′-TGCTGGCCTATGTCATGTGC-3′ and 5′-TCTGGATGTCGGAGGTAGCC-3′. Taqman MGB probe sequence for SGCA was 5′-CGGGAGGGAAGGCTGAAGAGAGACC-3′.

### Western immunoblotting

Whole-cell lysates of fibroblasts treated or not with 3 µM of thiostrepton or 30 nM of bortezomib were collected. Same cells extracts were treated with PNGase F (Biolabs) for 1 hour at 37 °C to remove N-linked oligosaccharides from glycoproteins. Proteins were extracted with RIPA lysis buffer (Thermo Scientific) supplemented with 1X Protease Inhibitor-Complete ULTRA tablets mini (Roche) and 1X Benzonase nuclease HC (Millipore) for 1 h at 4 °C. Protein concentration was measured using the Pierce BCA Protein Assay Kit (ThermoScientific) and the absorbance at 562 nm was evaluated using a Clariostar (BMG Labtech). A total of 50 μg of protein was loaded and run on NuPAGE Novex 4–12% Bis-Tris Protein Gels (Invitrogen) and transferred to nitrocellulose membranes (Thermo Scientific) following the manufacturer’s instructions. Membranes were then blocked in Odyssey blocking buffer (Li-Cor) for 1 hour at room temperature. Incubation with primary antibodies diluted in Odyssey blocking buffer was carried out at 4 °C overnight for the mouse anti-α-SG 1:200 (NCL-L-a-SARC, Novocastra, Leica) and for the rabbit anti-α-actin 1:200 (A2066, Sigma). Washing was carried out 4 times for 8 minutes at room temperature with TBS + 0.1% Tween20 and the membranes were incubated with a donkey anti-mouse antibody IRDye-680 (926–32222, Li-Cor) or a donkey anti-rabbit antibody IRDye-800 (926–32213, Li-Cor) at 1:10,000 in blocking buffer. Washing was carried out 4 times for 8 minutes at room temperature with TBS + 0.1% Tween20. Proteins were detected by fluorescence (Odyssey, Li-Cor) following the manufacturer’s instructions.

### Proteasomal activity assay

Fibroblasts and myoblasts were seeded in 384 well plates and treated with MG132 (1 µM) or various concentrations of bortezomib (100 pM–300 nM) and thiostrepton (10 nM–10 µM) for 12 hours. Proteasome-Glo™ chymotrypsin-like, trypsin-like or caspase-like cell-based assay reagents were added according to manufacturer instructions (Promega). Luminescence was read using a CLARIOstar® microplate reader (BMG Labtech).

### RNA sequencing and transcriptomic analysis

Total RNAs were isolated using the RNeasy Mini extraction kit (Qiagen) and quality of the RNAs was checked using a RNAnano chip (Agilent technologies) according to the manufacturer’ protocol. For each of the 12 samples, 50 ng of total RNA was reverse transcribed using the Ion AmpliSeq Transcriptome Human Gene Expression kit following the protocol of the manufacturer (Thermofisher Scientific). The cDNA libraries were amplified and barcoded using Ion AmpliSeq Transcriptome Human Gene Expression core panel and Ion Xpress Barcode Adapter (Thermofisher Scientific). The amplicons were quantified using Agilent High Sensitivity DNA kit before the samples were pooled in sets of eight. Emulsion PCR and Enrichment was performed on the Ion OT2 system Instrument using the Ion PI Hi-Q OT2 200 kit (Thermofisher Scientific). Samples were loaded on an Ion PI v3 Chip and sequenced on the Ion Proton System using Ion PI Hi-Q sequencing 200 kit chemistry (200 bp read length; Thermofisher Scientific). The sequencing reads (FASTQ files) were imported into the RNA-seq pipeline of Partek Flow software (v6 Partek Inc). Briefly, the pipeline consists of trimming of adapters and bases followed by a two-step alignment method. The sequencing reads were mapped on the Homo sapiens reference genome (hg19) with Tophat2 (v2.0.8). Then, the unaligned reads were realigned using Bowtie2 (v2.1.0). The resulting aligned reads from both the Tophat and the Bowtie2 steps were combined into one data node and annotated on RefSeq Release 84 using the Partek E/M algorithm. Gene abundances were quantified and expression levels were normalized on reads per million values (RPM). Differentially expressed genes were identified using Partek Gene Specific Analysis (GSA) when their FDR (False discovery rate) are lower than 0.01, the fold change are superior to 1.5 or inferior to −1.5 and the expression value in all sample are superior to 100 RPM. Biological interpretations of the filtered list of differentially expressed genes were performed using Enrichr software http://amp.pharm.mssm.edu/Enrichr/^36,37^. The heat map was generated by average linkage hierarchical clustering of the differentially expressed genes between THSP 3 µM and DMSO 0.1% (cutoff FDR ≤ 0.01 RPM ≥ 100), using Euclidean distance as the distance metric. Scaled expression values are color-coded according to the legend.

### Statistical analysis

Data are presented as means ± SD. Statistical analysis was performed using the Student’s t test. Statistical significance was considered for ***P ≤ 0.001. Curve-fitting, IC_50_ and EC_50_ determinations were performed using GraphPad Prism (v5.0.3).

## Supplementary information

Supplementary Information

## Data Availability

The datasets produced in this study are available in the following database: RNA-Seq data: Gene Expression Omnibus GSE11984.
