# Supplementary material for: Identification of thiostrepton as a pharmacological approach to rescue misfolded alpha-sarcoglycan mutant proteins from degradation
Source: Sci Rep. 2019 May 6;9:6915. doi: 10.1038/s41598-019-43399-w (PMC6502821; doi:10.1038/s41598-019-43399-w)
Supplement: Supplementary file 1 — Supplementary Information [file 41598_2019_43399_MOESM1_ESM.docx]

**Supplementary Information**

**Identification of thiostrepton as a pharmacological approach to rescue misfolded alpha-sarcoglycan mutant proteins from degradation**

Hoch Lucile, Henriques Sara F, Bruge Céline, Marsolier Justine, Benabides Manon, Bourg Nathalie, Tournois Johana, Mahé Gurvan, Morizur Lise, Jarrige Margot, Bigot Anne, Richard Isabelle and Nissan Xavier

**Supplemental Figure 1**


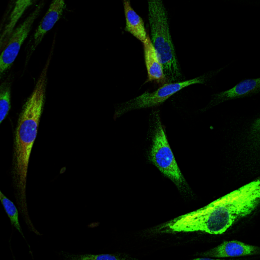

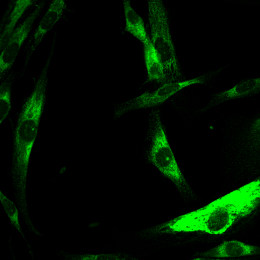

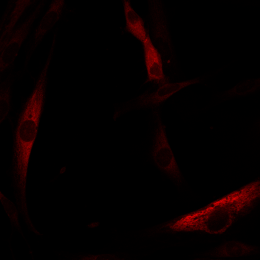

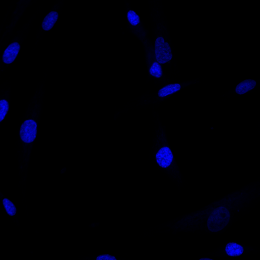

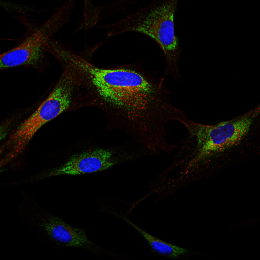

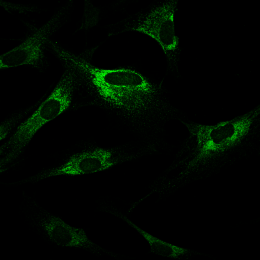

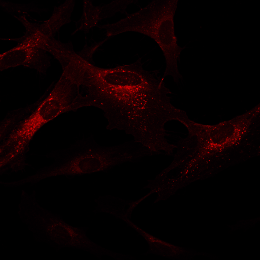

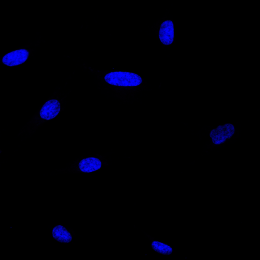


**A**

**WT-α-SGmCh**

**R77C-α-SGmCh**

**B**

**WT-α-SGmCh**

**R77C-α-SGmCh**

**Hoechst**

**mCherry**

**merged**

**20 µm**

**KDEL**

**SV40 pA**

**CMV**

**SGCA**

**linker**

**mCherry**

**SV40 pA**

**CMV**

**SGCA**

**linker**

**mCherry**

**R77C**

**Supplemental Figure 1. SGCAmCh fusion constructs and cellular characterization.** (**A**) Schematic representation of the lentiviruses used in the screening. The wildtype and mutated R77C SGCA coding sequence were fused to a mCherry sequence by a linker and expressed under the control of the cytomegalovirus (CMV) promoter. (**B**) Confocal images of mCherry fluorescent signal, KDEL and Hoechst staining under permeabilized condition in fibroblasts overexpressing the WT-α-SGmch or the R77C-α-SGmCh lentivirus. Scale bar = 50 µm.

**Supplemental Figure 2**


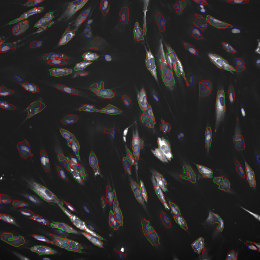

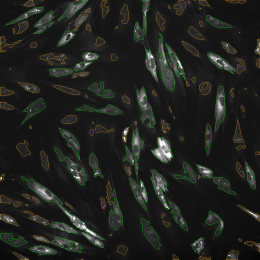

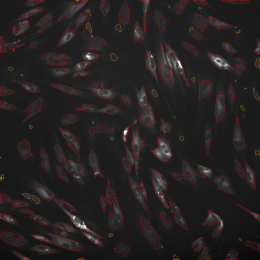

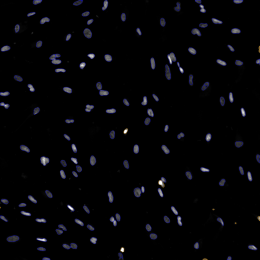

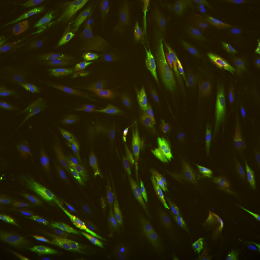

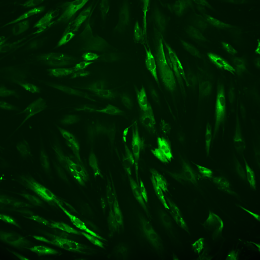

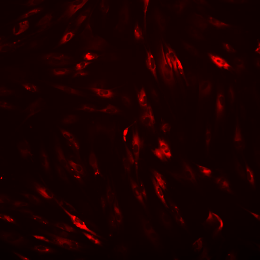

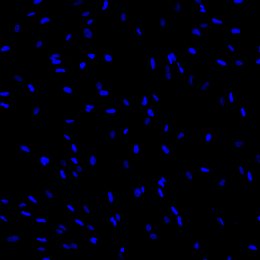


**A**

**Z’factor : 0.78**

**B**

**Hoechst**

**α-SG**

**mCherry**

**merged**

**staining**

**identification**

**colocalization**

**nuclei**

**identification**

**50 µm**

**tag**

**identification**

**Supplemental Figure 2. Development of a high throughput screening assay to quantify the effect of drugs on membrane α-SG expression in 384 well plates.** (**A**) mCherry fluorescent signal, membrane α-SG and Hoechst staining in fibroblasts overexpressing R77C-α-SGmCh and treated for 24 h of treatment with bortezomib 30 nM (top panels), and the corresponding masks images (bottom panels) allowing automated quantification of mCherry and membrane α-SG positive cells by colocalization. Scale bar = 50µm. (**B**) Determination of the Z’ factor following the screening protocol in 5 independent 384-well plates. Each dot represents in blue negative controls (DMSO 0.1 %) and in red positive controls (bortezomib 30 nM).

**Supplemental Figure 3**

**+ primary antibody**

**- primary antibody**

**Bortezomib**

**30 nM**

**Thiostrepton**

**3 µM**

**Pyrvinium**

**pamoate 5µM**

**α-SG**

**mCherry**

**α-SG**

**mCherry**

**50 µm**

**Supplemental Figure 3. Fluorescence interferences after pyrvinium pamoate treatment.** mCherry fluorescent signal and membrane α-SG staining in presence (left panels) or in absence (right panels) of α-SG primary antibody in fibroblasts overexpressing R77C-α-SGmCh and treated for 24 hours with bortezomib 30 nM, thiostrepton 3 µM or pyrvinium pamoate 5 µM. Detection of green-stained cells without α-SG primary antibody after pyrvinium pamoate treatment revealed fluorescence interferences of this drug. Scale bar = 50 µm.

**Supplemental Figure 4**

**A**

NT

THSP

BTZ

3,6%

0,1%

NT

THSP

BTZ

3,6%

0,1%

**WT**

**WT + DMSO**

**R77C**

**R77C + DMSO**


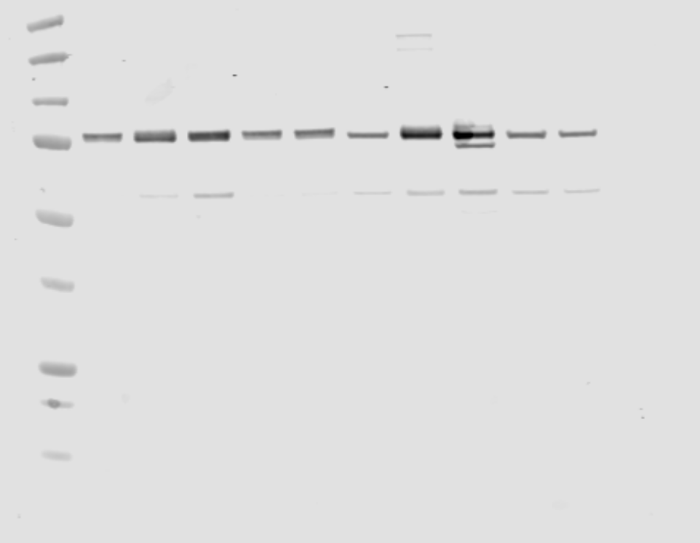


100kDa

75kDa

50kDa

37kDa

150kDa

200kDa

α-SG

α-SGmCh

**B**

NT

THSP

BTZ

3,6%

0,1%

NT

THSP

BTZ

3,6%

0,1%

**WT**

**WT + DMSO**

**R77C**

**R77C + DMSO**


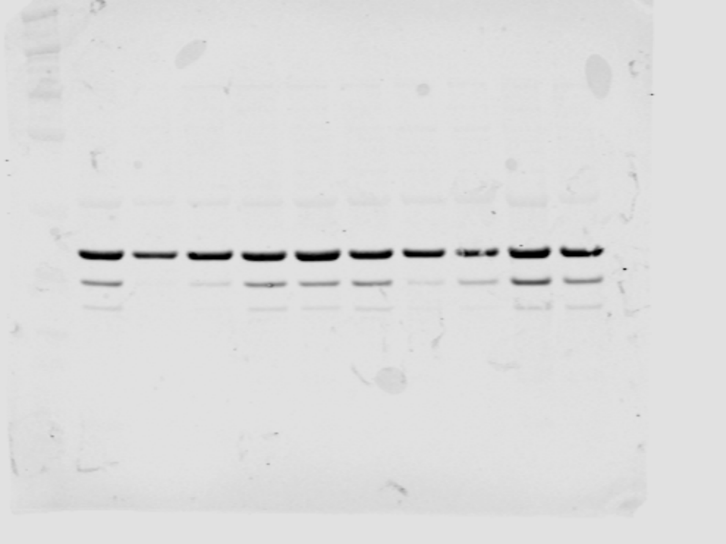


100kDa

75kDa

50kDa

37kDa

150kDa

200kDa

α-actin

**Supplemental Figure 4. Effect of thiostrepton and bortezomib on α-SG expression.** (**A**) Uncropped western blot analysis of α-SG expression in non‑treated (NT) fibroblasts overexpressing WT- or R77C-α-SGmCh or treated with DMSO (0,1% and 3,6%), thiostrepton (THSP, 3 µM) or bortezomib (BTZ, 30 nM) for 24 hours. (**B**) Uncropped western blot analysis of α-actin expression in the same cell extracts.

**Supplemental Figure 5**

-

+

-

-

-

+

+

+

**NT**

**THSP**

**BTZ**

**DMSO 3,6 %**

**A**

**PNGase F**


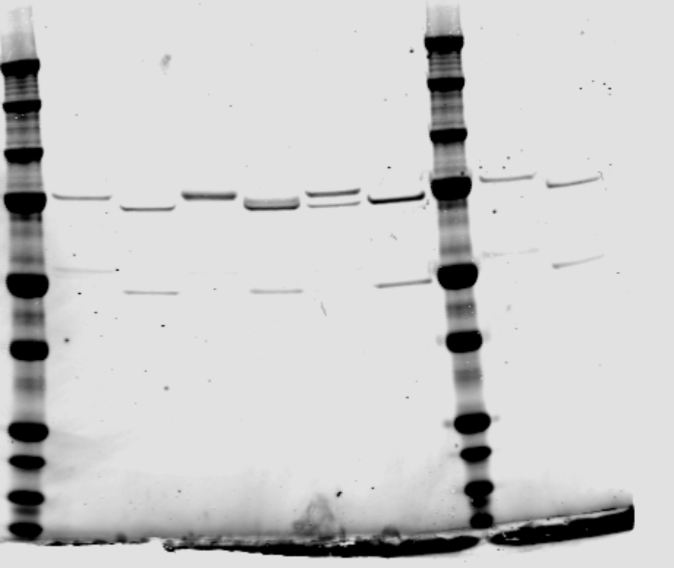


100kDa

75kDa

50kDa

37kDa

150kDa

200kDa

**α-SGmCh**

**α-SG**

-

+

-

-

-

+

+

+

**NT**

**THSP**

**BTZ**

**DMSO 3,6 %**

**B**

**PNGase F**


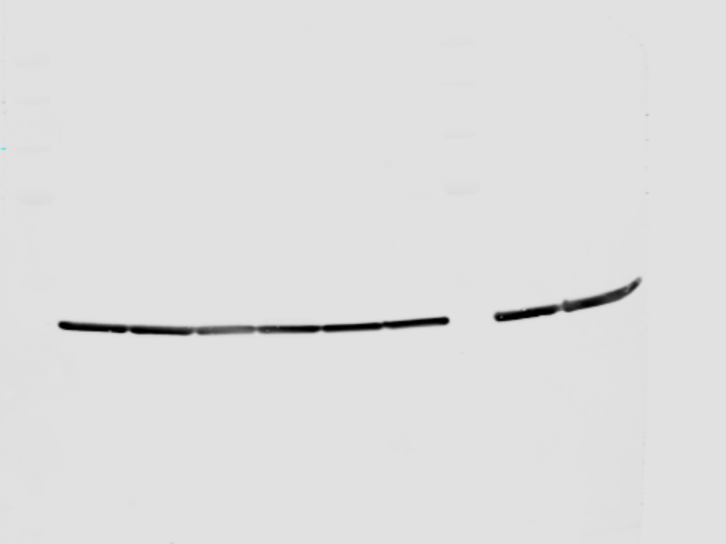


100kDa

75kDa

50kDa

37kDa

150kDa

200kDa

**α-actin**

**Supplemental Figure 5. Effect of thiostrepton and bortezomib on α-SG expression.** (**A**) Uncropped western blot analysis of α-SG expression in non‑treated (NT) fibroblasts overexpressing R77C-α-SGmCh or treated with DMSO (3,6%), thiostrepton (THSP, 3 µM) or bortezomib (BTZ, 30 nM) for 24 hours. Cell extracts were treated with PNGase F for 1 hour. (**B**) Uncropped western blot analysis of α-actin expression in the same cell extracts.

**Supplemental Figure 6**

**D**

**F**

**A**


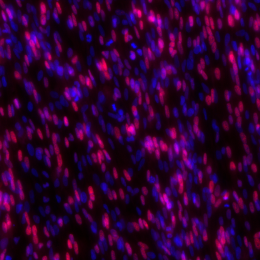

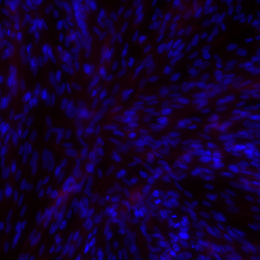

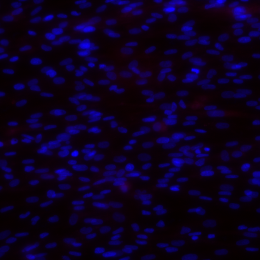

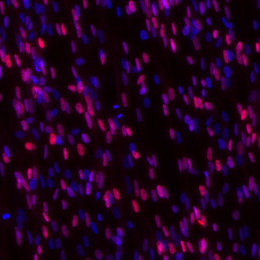


**MyoD / Hoechst**

**α-SG / Hoechst**

**WT Myoblasts**

**R77C Myoblasts**

**R77C iPSCs**

**OCT4**


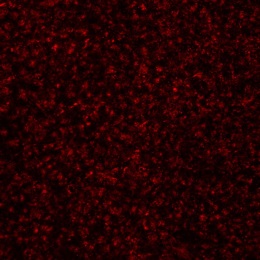


**SSEA4**

**NANOG**


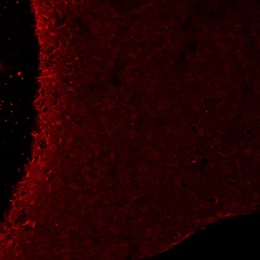


**TRA160**

**AP**

**MF20 / Hoechst**

**α-SG / Hoechst**

**WT Myotubes**

**R77C Myotubes**

**C**

**E**

**15 µm**

**50 µm**

**50 µm**

**50 µm**

**50 µm**

**250 µm**

**250 µm**

**B**

**50 µm**


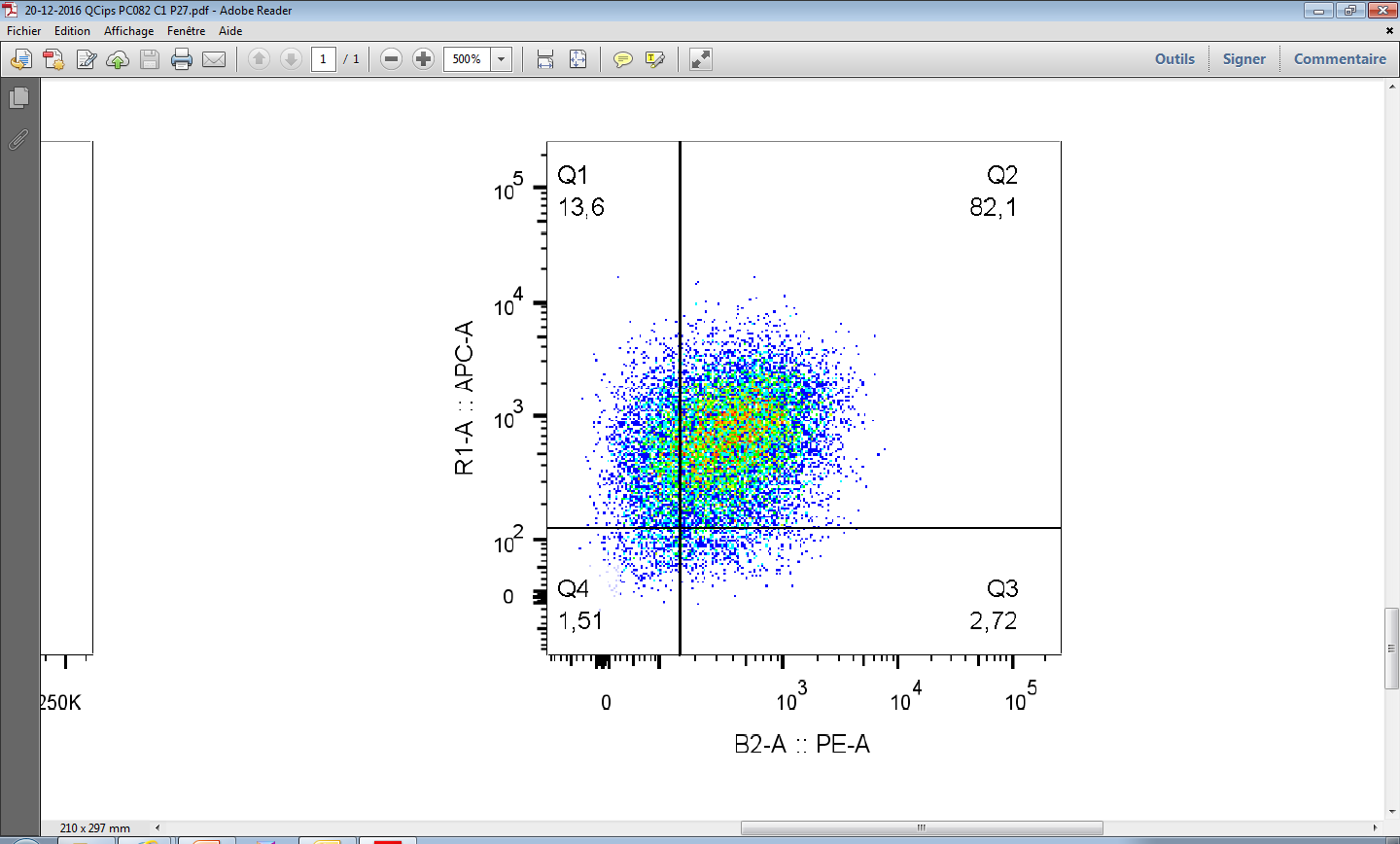


**TRA181**

**SSEA3**

**82.1 %**

**Supplemental Figure 6. Molecular characterization of R77C iPSCs and the differentiated muscle cells.** (**A**) Morphology (top panel) and measure of alkaline phosphatase (AP) activity (bottom panel) of R77C iPSCs. Scale bar = 250 µM. (**B**) Confocal images of SSEA4, TRA160, OCT4 and NANOG immunofluorescence staining. Scale bar = 50 µM. (**C**) Flow cytometry analysis of pluripotency and self-renewal markers TRA181 and SSEA3 in R77C iPSCs. (**D**) Immunostaining of MyoD and α-SG in WT and R77C iPSC-derived myoblasts. Scale bar = 50 µm. (**E**) Immunostaining of MF20 and α-SG in WT and R77C iPSC-derived myotubes. Scale bar = 15 µm. (**F**) Measure of SGCA gene expression by qPCR in immortalized or iPSC-derived myoblasts and myotubes. Data are normalized to iPSCs.

**Supplemental Table 1.** List of the 6 compounds identified as primary hits during the screening and the corresponding chemical structure, percentage of stimulation and percentage of cell viability.

| **Compound** | **Structure** | **% of stimulation**  **(mCherry/α-SG+ cells)** | **% of viability** |
| --- | --- | --- | --- |
| Pyrvinium Pamoate | 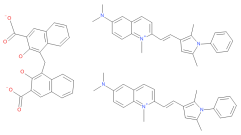 | 122.59 | 62.33 |
| Thiostrepton | 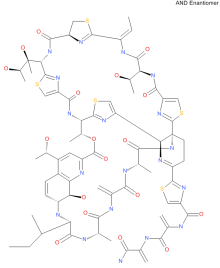 | 85.89 | 74.63 |
| Iodoacetamide | 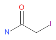 | 54.03 | 73.01 |
| BNTX |  | 45.93 | 79.50 |
| Brefeldin A | 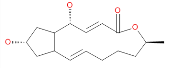 | 38.48 | 77.75 |
| BBMP | 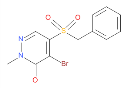 | 27.98 | 82.57 |

**Supplemental Table 2.** List of the primers used to introduce the R34H, R77C, I124T and V247M mutations.

| **Primer name** | **Primer Sequence** |
| --- | --- |
| R34H Forward | 5’-CACCCACTTGTGGGCCACGTCTTTGTGCACACC-3’ |
| R34H Reverse | 5’-GGTGTGCACAAAGACGTGGCCCACAAGTGGGTG-3’ |
| R77C Forward | 5’-GCCCCGGTGGCTCTGCTACACCCAGCGC-3’ |
| R77C Reverse | 5’-GCGCTGGGTGTAGCAGAGCCACCGGGGC-3’ |
| I124T Forward | 5’-CTGGTGCTGGAGACTGGGGACCCAGAA-3’ |
| I124T Reverse | 5’-TTCTGGGTCCCCAGTCTCCAGCACCAG-3’ |
| V247M Forward | 5’-GTTGACTGGTGCAATATGACCCTGGTGGATA-3’ |
| V247M Reverse | 5’-TATCCACCAGGGTCATATTGCACCAGTCAAC-3’ |
